# Dietary Habits, Awareness, and Knowledge among Polish Healthcare Providers and Healthcare Students

**DOI:** 10.3390/healthcare12191931

**Published:** 2024-09-26

**Authors:** Magdalena Kurnik-Łucka, Dominika Grońska, Iga Salwa, Julia Niedbałowska, Kamil Paweł Skowron, Katarzyna Anna Dyląg, Elżbieta Rząsa-Duran, Marcin Wojnarski, Agnieszka Pac, Krzysztof Gil

**Affiliations:** 1 Department of Pathophysiology, Jagiellonian University Medical College, Czysta 18, 31-121 Krakow, Poland; 2 Doctoral School of Medical and Health Sciences, Jagiellonian University Medical College, 31-008 Krakow, Poland; 3 Department of Epidemiology and Preventive Medicine, Jagiellonian University Medical College, 31-008 Krakow, Poland; 4 Pharmacists’ Chamber of Lesser Poland, 31-115 Krakow, Poland

**Keywords:** dietary awareness, nutrition, healthcare students, healthcare professionals, KomPAN questionnaire

## Abstract

**Background/Objectives**: Nutrition plays a significant role in preventive medicine, as up to 80% of chronic diseases could be avoided by adhering to healthy dietary principles. The aim of our study was to identify dietary habits, awareness, and nutrition knowledge in a random sample of Polish healthcare students and professionals. Methods: Initially, a total screened sample consisted of 1287 respondents, yet the actual response/participation rate was 634/1287. A validated questionnaire for the Polish population, the Dietary Habits and Nutrition Beliefs Questionnaire (KomPAN), was used for the assessment. **Results**: The majority of respondents were females (84.2% of medical students and 87.2% of healthcare professionals), current non-smokers (79.6% of medical students and 85.2% of healthcare professionals), and had a normal waist circumference (76.3% of medical students and 63.1% of healthcare professionals). Both clinical dieticians and students studying clinical dietetics received significantly different scores of dietary indexes (pro-healthy pHDI-10 and non-healthy nHDI-14) vs. respective groups of respondents. Both healthcare students and professionals who self-reported better nutritional knowledge indeed yielded significantly better results in the dietary indexes as well as nutritional knowledge scores. The nutrition knowledge score was positively correlated with pHDI-10 and negatively correlated with nHDI-14, BMI, age, and waist circumference. **Conclusions**: Our results illustrate lifestyle trends among Polish healthcare students and professionals, and such results should encourage the development of evidence-based dietary policies targeting healthcare providers. High-quality continuing nutrition education should be obligatorily provided to all healthcare providers to enhance their dietary awareness as well as ensure high quality of care and outcomes achieved by the Polish healthcare system.

## 1. Introduction

Up-to-date prospective research indicates that around 80% of chronic diseases (including diabetes and cancer) could be avoided by adhering to healthy dietary principles (a high intake of fruits, vegetables, and whole-grain bread with low meat consumption), smoking cessation, moderate exercise (at least 3.5 h per week or more of physical activity), and the maintenance of a healthy weight [1]. According to the Global Burden of Disease Study 2021, globally, smoking accounts for over 8 million annual deaths, excess salt/sodium intake for 1.8 million annual deaths, and insufficient physical activity for 830,000 annual deaths. The study estimated that over 91% of deaths in the European Union (EU) in 2019 resulted from non-communicable diseases (NCDs) such as cardiovascular diseases, diabetes, cancers, and chronic respiratory diseases [2]. For example, according to the International Diabetes Federation, the number of people living with diabetes in the EU is predicted to rise to 38 million by 2030, from approximately 33 million in 2010 [3]. Importantly, type 2 diabetes and obesity increasingly affect children, which requires dedicated management [4]. According to the European Commission Directorate-General for Health and Food Safety, a holistic approach to health promotion and disease prevention can reduce the burden of NCDs by as much as 70% [5].

Nutrition plays the most significant role in preventive medicine, and dietary behaviors are subject to modification. Healthcare professionals perform different roles to implement dietary recommendations, and all of them should have access to adequate continuing nutrition education [6]. They require evidence-based information together with implementation strategies that can be effectively utilized within the time constraints of their consultations. Healthcare providers acknowledge their important role in the provision of dietary advice to patients [7], yet nutrition knowledge and awareness does not guarantee satisfactory dietary habits among these professionals—a discussion about defining the relationship between theory and practice goes back to the ancient times of Aristotle and Plato [8]. While the dietary habits of a healthcare professional could influence their practice in providing nutritional interventions, direct evidence is scarce. Therefore, the aim of our study was to identify dietary habits and nutrition knowledge in a random sample of Polish healthcare students and professionals, as well as to assess the relationship between their nutrition knowledge and diet quality.

## 2. Materials and Methods

### 2.1. Study Sample, Recruitment, and Data Collection

The study sample was recruited between January 2021 and November 2023. The Dietary Habits and Nutrition Beliefs Questionnaire (KomPAN) [9,10,11] was used for the evaluation and was made available at Jagiellonian University Medical College domain “http://ankiety.cm-uj.krakow.pl”. The questionnaire was addressed to Polish medical students and Polish healthcare professionals with a valid professional license. Participation in the study was voluntary and anonymous. Information about the study was published on Polish healthcare students’ social media, the website of the Lesser Poland Chamber of Pharmacists, the Lesser Poland Chamber of Nurses and Midwives, the portal e-farmacja.pl, and additionally distributed among students and employees of the Jagiellonian University Medical College. The study protocol was approved by the Jagiellonian University Bioethics Committee (1072.6120.314.2020, issued on 25 November 2020).

### 2.2. The KomPAN Questionnaire

The Dietary Habits and Nutrition Beliefs Questionnaire (KomPAN) for people aged 15–65, designed by the Behavioral Nutrition Team, Committee of Human Nutrition, Polish Academy of Sciences, is a validated tool designed to assess nutrition habits in the Polish population [9,10,11]. The self-administered version of the questionnaire was used to collect food frequency consumption, nutrition beliefs to assess nutrition knowledge, and personal (weight, height, and waist circumference) together with elemental lifestyle (physical activity levels, sleeping time, and smoking practices) data.

### 2.3. Diet Quality and Nutrition Knowledge

Two diet quality scores (the Pro-Healthy Diet Index and the Non-Healthy Diet Index) were constructed as a sum of the daily frequency consumption of the selected food items and were determined strictly in accordance with the manual guide of the KomPAN questionnaire [9,10]. Respondents could choose one of six categories of consumption (further converted into values representing daily frequency consumption): never (0 times/day), 1–3 times a month (0.06 times/day), once a week (0.14 times/day), a few times a week (0.5 times/day), once a day (1.0 times/day), or a few times a day (2.0 times/day) to express the habitual consumption of different food items. The Pro-Healthy Diet Index (pHDI-10) included the sum of 10 food items representing pro-healthy food groups, and nHDI-14 included the sum of 14 food items representing unhealthy food groups. The sums were further converted to unify the total score range to a maximum of 100 points for each diet quality score, i.e., pHDI-10 = (100/20) × the sum of daily frequency consumption of 10 products and nHDI-14 = (100/28) × the sum of daily frequency consumption of 14 products. 

Nutrition knowledge was estimated using 25 statements (available as Supplementary Table S3) with 3 response categories: true, false, or unsure. The assessment was based on the predefined answer sheet in accordance with the manual guide of the KomPAN questionnaire, and for each statement, either 1 point or 0 points could be scored. The total nutrition knowledge score was calculated as a sum of points (0–25 points) and was grouped into the following categories: “insufficient” (0–8 points), “sufficient” (9–16 points), and “good” (17–25 points). Respondents were also asked to self-assess their overall nutrition knowledge and eating habits according to the following questions: “How do you rate your nutrition knowledge?” (insufficient, sufficient, good, very good) and “How do you rate your eating habits?” (very poor, poor, good, very good). 

Diet quality scores, consumption frequencies of the selected food items (median and interquartile range), and nutrition knowledge scores of the study respondents were also compiled with a sample from the healthy Polish population [12] and are presented as the Supplementary Material (Supplementary Table S2).

### 2.4. Sample Size and Statistical Analysis

The total study sample consisted of 1287 respondents. In the initial analysis, performed with Microsoft Excel for Mac 2011 software (version 14.7.2 Microsoft Corporation, Redmond, WA, USA, Jagiellonian University license), respondents were excluded for the following reasons: (1) incomplete data (*n* = 624); (2) failure to meet the predefined maximum age criterion, i.e., age over 65 years—for the KomPAN questionnaire (*n* = 3); (3) failed systemic verification of respondents’ reliability based on the KomPAN manual guide (*n* = 26). Consequently, 634 were included in the study.

Statistical analysis was performed with IBM SPSS Statistics (PS Imago Pro 10) licensed for Jagiellonian University. Distributions were evaluated for normality using the Shapiro–Wilk test. Distributions of all analyzed scores did not meet the criteria of normality, and thus the non-parametric Kruskal-Wallis test was applied to assess differences between the groups with the post-hoc Dunn’s test adjusted by the Bonferroni correction for multiple tests. The results of all tests were only considered statistically significant when *p*-values were below 0.05. Additionally, the Pearson correlation coefficient was calculated between dietary indexes (pHDI-10 and nHDI-14), nutrition knowledge score, age, body mass index, and waist circumference. Body mass index (BMI) values were calculated from height and weight, and the World Health Organization criteria, such as underweight < 18.5 kg/m^2^, normal 18.5–24.99 kg/m^2^, overweight ≥ 25 kg/m^2^, and obese ≥ 30 kg/m^2^, were used for the interpretation. A waist circumference (WC) below 80 cm in females and below 94 cm in males was interpreted as normal. 

We assessed the completeness of reporting with adherence to the STROBE checklist, including the title and abstract, introduction, methodology, results, discussion, and funding [13]. 

## 3. Results

### 3.1. Baseline Data

The sample used for calculations included 221 healthcare students and 413 healthcare professionals—the overall characteristics of both groups are given in Table 1 and Table 2. The majority of respondents were females (84.2% of medical students and 87.2% of working healthcare professionals), current non-smokers (79.6% of healthcare students and 85.2% of working healthcare professionals), and had a normal waist circumference (76.3% of healthcare students and 63.1% of working healthcare professionals). A BMI (kg/m^2^) ≥ 25 was found in 11.8% of healthcare students and 32.5% of healthcare professionals. The majority of respondents declared at least moderate recreational physical activity—69.2% of healthcare students and 71.8% of healthcare professionals (Supplementary Table S1).

The median age was 25 years (24–27) among healthcare students and 41 years (33–51) among healthcare professionals (Table 3).

### 3.2. Dietary Patterns and Nutrition Knowledge

Table 4, Table 5, Table 6 and Table 7 present median values and the interquartile range of the dietary indexes pHDI-10 and nHDI-14 among healthcare respondents (students and professionals, respectively). The non-parametric Kruskal-Wallis analysis revealed that both clinical dietitians and students studying clinical dietetics received significantly higher pHDI-10 scores and lower nHDI-14 scores vs. other healthcare students and professionals. At the same time, both medical doctors and students of medicine received the highest nHDI-14 score among respective respondents. 

Female healthcare students had significantly different dietary indexes (pHDI-10, *p* < 0.001, and nHDI-14, *p* = 0.011) vs. male respondents. Yet there were no differences in the dietary indexes with regard to waist circumference (elevated vs. normal WC) among the students. The Pearson correlation test showed that WC among healthcare students was significantly correlated with nHDI-14 (r = 0.331, *p* < 0.001) only. BMI among healthcare students was significantly correlated with nHDI-14 (r = 0.199, *p* = 0.004) and age (r = 0.168, *p* = 0.014), but not nutrition knowledge score or pHDI-10.

There were no sex-based differences in the dietary indexes among healthcare professionals. Healthcare professionals with elevated WC had worse outcomes in nHDI-14 (*p* = 0.01) compared to respondents with normal WC. The Pearson correlation test showed that WC among healthcare professionals was significantly correlated with nHDI-14 (r = 0.164, *p* = 0.006) and age (r = 0.151, *p* = 0.011), but not the nutrition knowledge score or pHDI-10. BMI values among healthcare professionals were significantly correlated with the nutrition knowledge score (r = −0.262, *p* < 0.001), nHDI-14 (r = 0.255, *p* < 0.001), and age (r = 0.363, *p* < 0.001) but not pHDI-10.

Among healthcare students, 67.9% or 7.7% reported their nutritional habits as “good” or “very good”, respectively (based on the following question: “Self-assessment of diet”—very poor, poor, good, very good). There was a significant variance in pHDI-10 (*p* < 0.001) and nHDI-14 (*p* < 0.001) between the groups, but not in nutrition knowledge scores in the self-assessment of nutritional habits. In post-hoc analysis, the “very good” group had significantly lower nHDI-14 values in comparison to the “good” (*p* = 0.022), “poor” (*p* < 0.001), and “very poor” (*p* = 0.003) groups. The “very good” group received significantly higher pHDI-10 values in comparison to the “good” (*p* = 0.029), “poor” (*p* < 0.001), and “very poor” (*p* = 0.008) groups.

Among healthcare professionals, 66.6% or 9.0% reported their nutritional habits as “good” or “very good”, respectively. There was a significant variance in nutrition knowledge test scores (*p* < 0.001), pHDI-10 (*p* < 0.001), and nHDI-14 (*p* < 0.001) between the groups in terms of the self-assessment of nutritional habits. In post-hoc analysis, the pHDI-10 values were significantly higher in the “very good” group (*p* < 0.001) compared to all other groups. The “very good” group scored significantly lower nHDI-14 values in comparison to the “good” (*p* = 0.05), “poor” (*p* < 0.001), and “very poor” (*p* = 0.018) groups. The “very good” group received significantly better nutrition knowledge scores in comparison to the “good” (*p* = 0.007), “poor” (*p* = 0.001), and “very poor” (*p* = 0.006) groups.

The frequency consumption of selected food items, which was used to calculate the dietary indexes, is presented in Table 8. Only 8.6% of healthcare students reported that they never snack, 70.6% reported not adding sugar or sweeteners to hot drinks, and 59.7% reported not adding salt to already cooked meals. Only 4.8% of healthcare professionals reported to never snack, 61.7% reported not adding sugar or sweeteners to hot drinks, and 60% reported not adding salt to already cooked meals (Supplementary Table S1). The median values and the interquartile ranges of pHDI-10, nHDI-14, and nutrition knowledge scores in comparison with a sample from the healthy Polish population [12] are given in Supplementary Table S2.

Among healthcare students, 71.5% of respondents scored “good” (a range of 17–25 points) in nutrition knowledge. The nutrition knowledge score was indifferent between respondents studying clinical dietetics in comparison with students of pharmacy (Table 9), and it was significantly different in comparison with students of medicine (*p* = 0.001) and clinical diagnostics (*p* = 0.001). The nutrition knowledge score among healthcare students was significantly correlated with nHDI-14 (r = −0.182, *p* = 0.007) and pHDI-10 (r = 0.325, *p* < 0.001). Among healthcare professionals, 64.2% of respondents had “good” nutrition knowledge. The nutrition knowledge score was significantly different across different healthcare professions (Table 10), being significantly higher (*p* < 0.001) among clinical dietitians in comparison with other groups. The nutrition knowledge score among healthcare professionals was also significantly correlated with nHDI-14 (r = −0.322, *p* < 0.001) and pHDI-10 (r = 0.223, *p* < 0.001).

At the same time, 44.8% and 11.3% of healthcare students self-assessed their nutritional knowledge (based on the following question: “Self-assessment of nutrition knowledge”—insufficient, sufficient, good, very good) as “good” or “very good”, respectively. Similarly, 41.2% and 12.8% of healthcare professionals self-assessed their nutritional knowledge as “good” or “very good”, respectively. Both healthcare students and professionals that self-reported better nutritional knowledge scored significantly better results in the dietary indexes as well as nutritional knowledge scores. Table 11 presents differences between all respondents (healthcare students and professionals, *n* = 634) based on their categorical nutrition knowledge evaluation. The nutrition knowledge score was positively correlated with pHDI-10 (r = 0.246, *p* < 0.00) and negatively correlated with nHDI-14 (r = −0.309, *p* < 0.001), BMI (r = −0.253, *p* < 0.001), age (r = −0.224, *p* < 0.001), and WC (r = −0.141, *p* = 0.004). The content of the nutrition knowledge evaluation (25 statements) according to the KomPAN questionnaire is available as Supplementary Table S3.

## 4. Discussion

Our study examined associations between a range of dietary and lifestyle behaviors as well as nutrition knowledge in a sample of Polish healthcare students and professionals. A validated, culture-specific tool with reliable psychometric properties was used for the assessment [8,9,10,14,15]. Our results revealed variable dietary patterns across health professions and stages of career (i.e., students vs. professionals). The analysis of the consumption frequencies of a priori selected food items, with regard to pHDI-10, revealed frequent (up to a few times a day) consumption of vegetables and fruits in both students and professionals, especially in comparison with the metabolically healthy general population [12]. Not surprisingly, clinical dietitians and students (especially females) studying clinical dietetics scored significantly higher values of the Pro-Healthy Diet Index (Table 6, Table 7, Table 9 and Table 10) and significantly lower values of the Non-Healthy Diet Index (Table 8 and Table 11). Medical doctors scored the highest values of the Non-Healthy Diet Index in comparison with other health professions. The consumption of fats, such as butter, vegetable oils, margarines, mixes of butter and margarines, cheese, tinned meat, red meat products, energy drinks, and alcoholic beverages, was generally comparable between healthcare professionals and the general population (Supplementary Table S2). On the other hand, regular consumption of dietary fats, such as vegetable oils rich in mono- and polyunsaturated fatty acids, should be consumed on a daily basis [16,17]. Matured cheese is also recommended, provided it is consumed in moderation [18] (especially alongside other fermented foods). Yet no information regarding the total quantities of food consumed, the quality of ingredients, the degree of processing, or the presence of unnecessary additives could be provided using a qualitative food frequency questionnaire such as the KomPAN, which limits the interpretation. The self-reported version is especially prone to inaccuracy, as well as systematic and random errors [19]. Snacking, for example, which was admitted by an ample amount of our respondents, is typical in other Western countries [20]. However, previous research has found snacking to be positively associated with increased energy intake and being overweight [21]. Previous reports have also revealed that snacking might be related to shift work; for example, nurses consumed fewer meals but had a higher snacking frequency on night shifts [22,23]. Still, Hulsegge et al. reported no differences between day and shift workers either in meal frequency, snacking frequency, or in the quality of snacks among healthcare workers [24]. Samhat et al. reported that night shift work during the night has a positive association between abnormal eating patterns and BMI among Lebanese nurses working night shifts; however, the increase in BMI was not related to eating habits [25]. Shift work was not assessed in the study, but it might negatively affect nutrition quality and account for weight gain, coronary artery disease, or metabolic syndrome [26]. Migdanis et al. concluded that shift-working healthcare professionals presented with disturbed eating behaviors, leading to the increased consumption of unhealthy food [27]. The values of the Non-Healthy Diet Index (nHDI-14) were significantly correlated with the waist circumference and BMI of our respondents, regardless of the KomPAN questionnaire imperfections. Thus, there is room for improvement in terms of the eating habits of all our healthcare respondents. Nutrition policymakers could encourage access to specific health-promoting snacks, such as nuts [28] or fruits and vegetables [29], that address nutrient insufficiencies together with motivational strategies on nutrition among the healthcare workforce [30]. The workplace has been identified as an important location for dietary interventions (up to 60% of daily food intake can occur in the workplace). However, evidence is limited in relation to healthcare professionals. Recent meta-analysis evaluated the effectiveness of such interventions, with results being inconclusive yet promising. For instance, interventions produced small to moderate increases in fruit and vegetable consumption, as well as significant decreases in fat and total energy intake [31].

Dietary awareness and knowledge are factors that improve eating behaviors, but they do not guarantee optimal dietary habits and satisfactory health outcomes. Previous studies reported a variable association between nutrition knowledge and food consumption [32,33,34,35]. Yet, in our study, nutrition knowledge scores were significantly correlated with the calculated dietary indexes among both students and healthcare professionals. Knowledge itself does not stimulate change, but positive eating habits may result from self-recognition of the importance of healthy dietary habits [36]. Our results indicate that worse self-reported nutritional knowledge was also associated with lower nutritional knowledge scores, as well as inadequate dietary indexes. Age should be taken into consideration in this context, as nutrition knowledge scores were negatively correlated with age and nutrition knowledge scores were significantly higher in healthcare students as compared to healthcare professionals. Nurses, whose median age was the highest, received the lowest nutrition knowledge scores. Thus, our results emphasize the need for life-long nutrition education for all healthcare professionals [37] at all stages of their career. Globally, physicians recall receiving limited or inadequate nutrition education in medical school [38,39,40,41,42], yet they remain the most credible source for dietary information according to patients [40]. Our results highlight a strong need for expanding the opportunities for and involvement of registered dietitians to improve Polish health outcomes, as both healthcare professionals and students presented with the highest nutrition knowledge. Pharmacists should also be engaged in monitoring and education on lifestyle modifications as an adjunct to pharmacotherapy [43], which is justified by their satisfactory nutrition knowledge and dietary awareness revealed in our study. Thus, ideally, a widely available collaborative service involving a range of healthcare professionals would provide more holistic patient care.

## 5. Limitations

Self-reported dietary assessments, such as ours, are prone to underreporting bias [44] and errors, such as a lack of daily nutrient and caloric intake [19]. An uneven number of male and female respondents, as well as an inconsistent number of representatives of different medical specialties, altered the statistical integrity and reduced the accuracy of the assessment. Yet the length and the content (for example, the calculated dietary indexes are based on an omnivore diet only) of the questionnaire might have discouraged respondents and resulted in the meaningfully diminished response rate. An interest toward health evaluations is characteristic for people with higher nutrition knowledge [45] and food literacy [46] too.

## 6. Conclusions

Our results illustrate nutrition trends among Polish healthcare students and professionals, and such results should encourage the development of specific evidence-based dietary policies in healthcare institutions dedicated to both patients and the workforce. Nutrition is a cornerstone of modern medicine, and a personalized approach to nutrition provides essential building blocks for cellular function, immune system strength, and organ performance. Registered dietitians should be able to more actively participate in the process of patient care, which could ease the overwhelming burden of physicians in Poland. Pharmacists, with around-the-clock accessibility and acceptable health knowledge [42], should also be able to overstep their traditional function of dispensing and distributing medicines and provide population-based nutrition counseling as an integral part of effective pharmacotherapy. High-quality continuing nutrition education should be obligatorily provided to all healthcare providers to enhance their own dietary awareness as well as ensure the high quality of care and outcomes achieved by the Polish healthcare system.

## Figures and Tables

**Table 1:** Characteristics of the students’ respondents.

		Type of Healthcare Studies			
		Medicine (*n* = 64)	Clinical Dietetics (*n* = 64)	Clinical Diagnostics (*n* = 47)	Other * (*n* = 46)
Sex	Females	40 (62.5%)	58 (90.6%)	46 (97.9%)	42 (91.3%)
	Males	24 (37.5%)	6 (9.4%)	1 (2.1%)	4 (8.7%)
Smoking	No	63 (98.4%)	58 (90.5%)	46 (97.9%)	44 (95.7%)
	Yes	1 (1.6%)	6 (9.4%)	1 (2.1%)	2 (4.3%)
	Yes, used to smoke **	11 (17.5%)	20 (31.3%)	2 (4.3%)	11 (25.0%)

* Other includes: pharmacy students (*n* = 26), physiotherapy students (*n* = 3), nursing students (*n* = 2), midwifery students (*n* = 1), clinical psychology students (*n* = 7), and students from other, unspecified medical majors (*n* = 7). ** in brackets % of those who do not smoke now.

**Table 2:** Characteristics of the healthcare professionals’ respondents.

		Healthcare Professionals				
		Pharmacists (*n* = 191)	Nurses (*n* = 96)	Clinical Dietitians (*n* = 44)	Medical Doctors (*n* = 28)	Other * (*n* = 54)
Sex	Female	161 (84.3%)	94 (97.9%)	39 (88.6%)	17 (60.7%)	49 (90.7%)
	Male	30 (15.7%)	2 (2.1%)	5 (11.4%)	11 (39.3%)	5 (9.3%)
Smoking	No	185 (96.9%)	67 (69.8%)	38 (86.4%)	20 (71.4%)	43 (79.6%)
	Yes	6 (3.1%)	29 (30.2%)	6 (13.6%)	8 (28.6%)	11 (20.4%)
	Yes, used to smoke **	41 (22.2%)	43 (44.8%)	11 (25.0%)	9 (32.1%)	17 (31.5%)

* Other includes: dentists (*n* = 1), physiotherapists (*n* = 5), clinical diagnosticians (*n* = 5), midwives (*n* = 10), clinical psychologists (*n* = 12), paramedics (*n* = 2), and other, unspecified medical professions (*n* = 19). ** in brackets % of those who do not smoke now.

**Table 3:** Age groups among healthcare professionals.

		Healthcare Professionals				
		Pharmacists (*n* = 191)	Nurses (*n* = 96)	Clinical Dietitians (*n* = 44)	Medical Doctors (*n* = 28)	Other * (*n* = 54)
Age groups	20–34 years	17 (8.9%)	17 (17.7%)	33 (75.0%)	11 (39.3%)	107 (25.9%)
	35–49 years	108 (56.5%)	36 (37.5%)	11 (25.0%)	5 (17.9%)	181 (43.8%)
	Above 50 years	66 (34.6%)	43 (44.8%)	0 (0.0%)	12 (42.9%)	125 (30.3%)
Age (median and interquartile range)		45 (39–53)	48.5 (36.6–55)	31 (28–34.75)	40 (29–55)	34 (29–41)

* Other includes: dentists (*n* = 1), physiotherapists (*n* = 5), clinical diagnosticians (*n* = 5), midwives (*n* = 10), clinical psychologists (*n* = 12), paramedics (*n* = 2), and other, unspecified medical professions (*n* = 19).

**Table 4:** Pro-Healthy Diet Index (pHDI-10) among healthcare students (median and interquartile range).

		pHDI among Healthcare Students		*p*-Value
		Median	Q_25_–Q_75_	
Type of healthcare studies	Medicine (*n* = 64)	26.65	15.45–33.00	<0.001
	Clinical dietetics (*n* = 64)	38.05	30.20–43.90	
	Clinical diagnostics (*n* = 47)	24.10	15.90–34.50	
	Other * (*n* = 46)	28.45	23.80–34.90	

* Other includes: pharmacy students (*n* = 26), physiotherapy students (*n* = 4), nursing students (*n* = 2), midwifery students (*n* = 2), clinical psychology students (*n* = 7), and students from other, unspecified medical majors (*n* = 7).

**Table 5:** Non-Healthy Diet Index (nHDI-14) among healthcare students (median and interquartile range).

		nHDI among Healthcare Students		*p*-Value
		Median	Q_25_–Q_75_	
Type of healthcare studies	Medicine (*n* = 64)	14.50	10.61–18.04	0.003
	Clinical dietetics (*n* = 64)	9.54	6.43–14.43	
	Clinical diagnostics (*n* = 47)	12.14	9.64–16.14	
	Other * (*n* = 46)	10.89	8.07–17.43	

* Other includes: pharmacy students (*n* = 26), physiotherapy students (*n* = 4), nursing students (*n* = 2), midwifery students (*n* = 2), clinical psychology students (*n* = 7), and students from other, unspecified medical majors (*n* = 7).

**Table 6:** Pro-Healthy Diet Index (pHDI-10) among healthcare professionals (median and interquartile range).

		pHDI among Healthcare Professionals		
		Median	Q_25_–Q_75_	*p*-Value
Healthcare professionals	Pharmacists (*n* = 191)	29.20	21.50–36.70	0.014
	Nurses (*n* = 96)	26.35	20.35–37.50	
	Clinical dietitians (*n* = 44)	40.85	29.88–48.73	
	Medical doctors (*n*= 28)	33.30	25.10–39.43	
	Other * (*n* = 54)	24.15	16.75–32.33	

* Other includes: dentists (*n* = 1), physiotherapists (*n* = 5), clinical diagnosticians (*n* = 5), midwives (*n* = 10), clinical psychologists (*n* = 12), paramedics (*n* = 2), and other, unspecified medical professions (*n* = 19).

**Table 7:** Non-Healthy Diet Index (nHDI) among healthcare professionals (median and interquartile range).

		nHDI among Healthcare Professionals		
		Median	Q_25_–Q_75_	*p*-Value
Healthcare professionals	Pharmacists (*n* = 191)	14.40	8.70–20.30	<0.001
	Nurses (*n* = 96)	15.15	9.70–21.30	
	Clinical dietitians (*n* = 44)	8.55	4.30–13.05	
	Medical doctors (*n* = 28)	18.95	11.6–26.78	
	Other * (*n* = 54)	15.25	10.53–19.03	

* Other includes: dentists (*n* = 1), physiotherapists (*n* = 5), clinical diagnosticians (*n* = 5), midwives (*n* = 10), clinical psychologists (*n* = 12), paramedics (*n* = 2), and other, unspecified medical professions (*n* = 19).

**Table 8:** Diet quality scores (in times/day) assessed as Pro-Healthy Diet Index (pHDI-10, range 0–100 points), Non-Healthy Diet Index (nHDI-14, range 0–100 points), and consumption frequencies of the selected food items (median and interquartile ranges). Nutrition knowledge evaluation based on 25 questions (median and interquartile range): insufficient (0–8 points), sufficient (9–16 points), good (17–25 points).

	Healthcare Students (*n* = 221)		Healthcare Professionals (*n* = 413)		*p*-Value
	Median	Q_25_–Q_75_	Median	Q_25_–Q_75_	
pHDI-10	30.0	20.5–38.5	29.2	21.4–38.1	0.743
Wholemeal bread	0.50	0.06–1.00	0.5	0.14–1.0	0.593
Buckwheat, oats, whole grain pasta, and other coarse-ground groats	0.50	0.14–0.50	0.5	0.06–0.5	0.005
Milk	0.50	0.06–1.00	0.5	0.06–1.0	0.472
Fermented milk beverages	0.50	0.14–0.50	0.5	0.14–1.0	0.029
Fresh cheese (cottage cheese) curd products	0.14	0.06–0.50	0.5	0.14–1.0	<0.001
White meat products	0.50	0.14–0.50	0.5	0.14–0.5	0.913
Fish	0.06	0.06–0.14	0.14	0.06–0.14	0.011
Pulse-based food products	0.14	0.06–0.50	0.14	0.06–0.32	0.105
Fruits	1.00	0.50–2.00	1.0	0.5–2.0	0.575
Vegetables	2.00	1.00–2.00	1.0	1.0–2.0	0.031
nHDI-14	11.9	8.21–17.0	14.4	8.8–20.3	<0.001
White bread	0.50	0.14–1.00	0.5	0.14–1.0	0.123
White rice, white pasta, fine-ground groats	0.50	0.14–0.50	0.14	0.06–0.5	0.033
Fast foods	0.06	0.06–0.14	0.06	0.06–0.14	0.181
Fried foods	0.14	0.06–0.50	0.14	0.06–0.5	0.144
Butter, vegetable oils, margarines, mixes of butter and margarines	0.14	0.06–0.50	0.5	0.06–1.0	<0.001
Lard (as an addition to bread or dishes, for frying, baking, etc.)	0.00	0.00–0.00	0.0	0.0–0.06	<0.001
Cheese	0.50	0.14–0.50	0.5	0.14–0.5	0.861
Cold meats, smoked sausages, hot dogs	0.14	0.06–0.50	0.5	0.06–0.5	0.002
Red meat products	0.06	0.06–0.14	0.14	0.06–0.5	<0.001
Sweets	0.50	0.14–0.50	0.5	0.14–1.0	0.576
Tinned meat	0.00	0.00–0.00	0.0	0.0–0.06	<0.001
Sweetened beverages	0.06	0.00–0.06	0.06	0.0–0.14	0.401
Energy drinks	0.00	0.00–0.06	0.0	0.0–0.06	0.005
Alcoholic beverages	0.06	0.06–0.14	0.06	0.06–0.14	0.062
Nutrition knowledge	19	16–21	17	14–20	<0.001

**Table 9:** Nutrition knowledge among healthcare students (median and interquartile range).

		Nutrition Knowledge Score		*p*-Value
		Median	Q_25_–Q_75_	
Type of healthcare studies	Medicine (*n* = 64)	18.0	15–19.0	*p* < 0.001
	Clinical dietetics (*n* = 64)	20.5	18–22.0	
	Clinical diagnostics (*n* = 47)	17.0	15–19.5	
	Other * (*n* = 46)	18.0	15–21.0	

* Other includes: pharmacy students (*n* = 26), physiotherapy students (*n* = 4), nursing students (*n* = 2), midwifery students (*n* = 2), clinical psychology students (*n* = 7), and students from other, unspecified medical majors (*n* = 7).

**Table 10:** Nutrition knowledge among healthcare professionals (median and interquartile range).

		Nutrition Knowledge Score		*p*-Value
		Median	Q_25_–Q_75_	
Healthcare professionals	Pharmacists (*n* = 191)	18.0	16–20.0	<0.001
	Nurses (*n* = 96)	14.0	11.5–17.0	
	Clinical dietitians (*n* = 44)	21.0	20–22.5	
	Medical doctors (*n*= 28)	15.5	11–18.0	
	Other * (*n* = 54)	14.5	12–17.0	

* Other includes: dentists (*n* = 1), physiotherapists (*n* = 5), clinical diagnosticians (*n* = 5), midwives (*n* = 10), clinical psychologists (*n* = 12), paramedics (*n* = 2), and other, unspecified medical professions (*n* = 19).

**Table 11:** Categorical nutrition knowledge evaluation based on 25 questions with regard to age [years], BMI [kg/m^2^], WC [cm], and the dietary indexes: pHDI-10 and nHDI-14 [0–100 points].

Nutrition Knowledge Score							*p*-Value
	Good (17–25 Points)		Sufficient (9–16 Points)		Insufficient (0–8 Points)		
	Median	Mean ± SD	Median	Mean ± SD	Median	Mean ± SD	
Age (*n* = 634)	31	34.6 ± 10.8	40	39.9 ± 12.8	36	39.5 ±11.3	<0.001 ^a^
BMI (*n* = 619) *	21.6	22.7 ± 4.3	23.4	24.5 ± 4.7	25.1	25.9 ± 5.2	<0.001 ^b^
WC (*n* = 420) *	76	78.6 ± 12.8	80	81.8 ± 13.9	83	82.6 ±13.6	0.018 ^c^
pHDI-10 (*n* = 634)	31.6	31.5 ± 12.2	26.7	27.5 ± 11.6	28.6	29.0 ± 11.2	<0.001 ^d^
nHDI-14 (*n* = 634)	12.1	13.2 ± 7.0	15.4	16.2 ± 8.6	22.5	25.8 ± 14.6	<0.001 ^e^

* Information regarding weight, height, and waist circumference was given voluntarily. Post-hoc analysis: ^a^—good vs. sufficient (*p* < 0.001). ^b^—good vs. sufficient (*p* < 0.001), good vs. insufficient (*p* = 0.007). ^c^—good vs. sufficient (*p* = 0.024). ^d^—good vs. sufficient (*p* < 0.001). ^e^—good vs. sufficient (*p* < 0.001), good vs. insufficient (*p* < 0.001), sufficient vs. sufficient (*p* = 0.014).

## Data Availability

Inquiries can be directed to the corresponding author.

## Supplementary Materials

The following supporting information can be downloaded at: https://www.mdpi.com/article/10.3390/healthcare12191931/s1, Table S1: Sample characteristics by lifestyle variables according to sex and professional status; Table S2: Diet quality scores (in times/day) expressed as pro-Healthy-Diet-Index (pHDI), non-Healthy-Diet-Index (nHDI), and consumption frequencies of the selected food items (median and interquartile range) in comparison with the Polish population data [12]; Table S3: The content of the nutrition knowledge test according to the KomPAN questionnaire [9,10,11].
