# Supplementary material for: Dietary Habits, Awareness, and Knowledge among Polish Healthcare Providers and Healthcare Students
Source: Healthcare (Basel). 2024 Sep 26;12(19):1931. doi: 10.3390/healthcare12191931 (PMC11475298; doi:10.3390/healthcare12191931)
Supplement: Supplementary file 1 [file healthcare-12-01931-s001.zip › healthcare-3194490-supplementary.pdf]

Dietary habits, awareness and knowledge among Polish healthcare providers

Supplementary Table S1. Sample characteristics by lifestyle variables according to sex and professional status.

	Healthcare professionals		Medical students	
	Females (n=360)	Males (n=53)	Females (n=186)	Males (n=35)
**Waist circumference**[a]				
Within normal range	153 (42.5)	25 (47.2)	96 (51.6)	17 (48.6)
Above normal range	94 (26.1)	10 (18.9)	20 (10.8)	5 (14.3)
No data	113 (31.4)	18 (33.9)	70 (37.6)	13 (37.1)
**Sleep time on weekdays (hours/day)**[b]				
≤6	133 (36.9)	19 (35.8)	58 (31.2)	14 (40.0)
7-8	217 (60.3)	31 (58.5)	124 (66.7)	20 (57.1)
≥9	10 (2.8)	3 (5.7)	4 (2.1)	1 (2.9)
**Sleep time on weekends (hours/day)**[c]				
≤6	42 (11.6)	5 (9.5)	13 (6.9)	1 (2.9)
7-8	236 (65.6)	36 (67.9)	110 (59.2)	23 (65.7)
≥9	82 (22.8)	12 (22.6)	63 (33.9)	11 (31.4)
**Recreational physical activity**[d]				
Low	108 (30.0)	11 (20.8)	57 (30.6)	11 (31.4)
Moderate	186 (51.7)	28 (52.8)	98 (52.7)	10 (28.6)
High	66 (18.3)	14 (26.4)	31 (16.7)	14 (40.0)
**Eating away from home**[e]				
Never	82 (22.8)	10 (18.9)	22 (11.8)	5 (14.3)
1–3 times per month	161 (44.7)	19 (35.8)	84 (45.2)	18 (51.4)
Once a week	78 (21.7)	10 (18.9)	59 (31.7)	7 (20.0)
Few times per week	35 (9.7)	12 (22.6)	21 (24.4)	3 (8.6)
Once a day	3 (0.8)	2 (3.8)	0 (0)	2 (5.7)
Few times per day	1 (0.3)	0 (0)	0 (0)	0 (0)
**Self-assessed nutritional knowledge**[f]				
Insufficient	23 (6.4)	3 (5.7)	16 8.6)	3 (8.6)
Sufficient	146 (40.6)	18 (33.9)	65 (34.9)	13 (37.1)
Good	145 (40.3)	25 (47.2)	84 (45.2)	15 (42.9)
Very good	46 (12.7)	7 (13.2)	21 (24.4)	4 (11.4)
**Self-assessed dietary habits**[g]				
Very poor	6 (1.7)	1 (1.8)	5 (2.7)	1 (2.9)
Poor	81 (22.5)	13 (24.5)	37 (19.9)	11 (31.4)
Good	241 (66.9)	34 (64.2)	130 (69.9)	20 (57.1)
Very good	32 (8.9)	5 (9.5)	14 (7.5)	3 (8.6)
**Adding extra salt to food after cooking**[h]				
Yes	140 (38.9)	25 (47.2)	75 (40.3)	14 (40.0)
No	220 (61.1)	28 (52.8)	111 (59.7)	21 (4.2)
**Adding sugar to hot drinks**[i]				
Yes (sugar or honey)	119 (33.1)	19 (35.8)	45 (24.2)	8 (22.9)
Yes (artificial sweeteners)	15 (4.1)	5 (9.5)	8 (4.3)	4 (11.4)
No	226 (62.8)	29 (54.7)	133 (71.5)	23 (65.7)
**Snacking**[j]				
Never	15 (4.2)	5 (9.5)	18 (9.7)	1 (2.9)
1–3 times a month	27 (7.5)	4 (7.5)	14 (7.5)	1 (2.9)
Once a week	51 (14.2)	11 (20.7)	37 (19.9)	6 (17.1)
Few times per week	144 (40.0)	15 (28.3)	74 (39.8)	14 (40.0)
Once a day	64 (17.8)	9 (17.0)	25 (13.4)	4 (11.4)
Few times per day	59 (16.3)	9 (17.0)	18 (9.7)	9 (25.7)

Supplementary Table S2. Diet quality scores (in times/day) expressed as pro-Healthy-Diet-Index (pHDI), non-Healthy-Diet-Index (nHDI), and consumption frequencies of the selected food items (median and interquartile range) in comparison with the Polish population data [12].

	Medical students (n = 221)		Healthcare professionals (n = 413)		Polish population data (n=565)	
	Median	Q_{25}-Q_{75}	Median	Q_{25}-Q_{75}	Median	Q_{25}-Q_{75}
**pHDI-10**	**6.0**	**4.1-7.7**	**5.84**	**4.3-7.6**	**3.9**	**2.9–5.5**
Whole-meal bread	0.50	0.06-1.00	0.5	0.14-1.0	0.14	0.06–0.50
Buckwheat, oats, whole grain pasta and other coarse-ground groats	0.50	0.14-0.50	0.5	0.06-0.5	0.14	0.06–0.50
Milk	0.50	0,06-1.00	0.5	0.06-1.0	0.50	0.14–1.00
Fermented milk beverages	0.50	0.14-0.50	0.5	0.14-1.0	0.14	0.06–0.50
Fresh cheese (cottage cheese) curd products	0.14	0.06-0.50	0.5	0.14-1.0	0.14	0.06–0.50
White meat products	0.50	0.14-0.50	0.5	0.14-0.5	0.50	0.14–0.50
Fish	0.06	0.06-0.14	0.14	0.06-0.14	0.06	0.06–1.00
Pulse-based food products	0.14	0.06-0.50	0.14	0.06-0.32	0.06	0.06–0.06
Fruits	1.00	0.50-2.00	1.0	0.5-2.0	0.50	0.50–1.00
Vegetables	2.00	1.00-2.00	1.0	1.0-2.0	0.50	0.50–1.00
**nHDI-14**	**3.3**	**2.3-4.8**	**4.0**	**2.5-5.7**	**4.5**	**3.1–6.1**
White bread	0.50	0.14-1.00	0.5	0.14-1.0	0.50	0.50–1.00
White rice, white pasta, fine-ground groats	0.50	0.14-0.50	0.14	0.06-0.5	0.14	0.06–0.50
Fast foods	0.06	0.06-0.14	0.06	0.06-0.14	0.06	0.06–0.14
Fried foods	0.14	0.06-0.50	0.14	0.06-0.5	0.50	0.14–0.50
Butter, vegetable oils, margarines, mixes of butter and margarines	0.14	0.06-0.50	0.5	0.06-1.0	0.50	0.06–1.00
Lard (as an addition to bread or dishes, for frying, baking, etc.)	0.00	0.00-0.00	0.0	0.0-0.06	0.00	0.00–0.06
Cheese	0.50	0.14-0.50	0.5	0.14-0.5	0.50	0.14–0.50
Cold meats, smoked sausages, hot-dogs	0.14	0.06-0.50	0.5	0.06-0.5	0.50	0.14–0.50
Red meat products	0.06	0.06-0.14	0.14	0.06-0.5	0.14	0.06–0.50
Sweets	0.50	0.14-0.50	0.5	0.14-1.0	0.50	0.14–0.50
Tinned meat	0.00	0.00-0.00	0.0	0.0-0.06	0.00	0.00–0.06
Sweetened beverages	0.06	0.00-0.06	0.06	0.0-0.14	0.06	0.06–0.50
Energy drinks	0.00	0.00-0.06	0.0	0.0-0.06	0.00	0.00–0.06
Alcoholic beverages	0.06	0.06-0.14	0.06	0.06-0.14	0.06	0.06–0.14

Supplementary Table S3. The content of the nutrition knowledge test according to the KomPAN questionnaire [9-11].

Nutrition knowledge statements
It is enough to eat whole grains/cereals once a day.
Only children and adolescents should drink milk.
Fruit and/or vegetables should be consumed with every meal.
Consumption of moldy bread can result in food poisoning caused by Salmonella.
High intakes of salt protect from hypertension.
Limiting high-fat foods in everyday diet is protective against cardiovascular diseases.
Frequent consumption of oily fish contributes to atherosclerosis.
Frequent consumption of grilled meats contributes to the onset of cancer.
Vegetarian diet increases the risk of anemia.
Bio-yoghurts contain beneficial gut bacteria.
Vegetable oils and olive oil contain a high amount of cholesterol.
Whole-meal bread has more fiber than white bread.
Fruit and vegetables are a source of 'empty calories'.
Butter and fortified margarines have high content of vitamin A and D.
Cheese is a better source of calcium than cottage cheese.
Offal has high amounts of 'bad' cholesterol - LDL.
In a healthy diet, complex carbohydrates should be replaced with simple sugars.
In a balanced diet, proteins should be the main source of energy.
Inadequate intakes of vitamin PP can cause skin inflammation and diarrhea.
Sun exposure increases the synthesis of vitamin D in the human body.
Phosphorus is a component of neural tissue.
The ratio of calcium to phosphorus in a healthy diet should be 1:1.
Consumption of fruit with high content of vitamin C increases bioavailability of iron.
Starting cooking vegetables in cold water helps to preserve the nutrients.
Sweets and animal fats are particularly high nutrient dense foods.
